# Supplementary material for: In vivo patch-clamp recordings reveal distinct subthreshold signatures and threshold dynamics of midbrain dopamine neurons
Source: Nat Commun. 2020 Dec 8;11:6286. doi: 10.1038/s41467-020-20041-2 (PMC7722714; doi:10.1038/s41467-020-20041-2)
Supplement: Supplementary file 1 — Supplementary Information [file 41467_2020_20041_MOESM1_ESM.pdf]

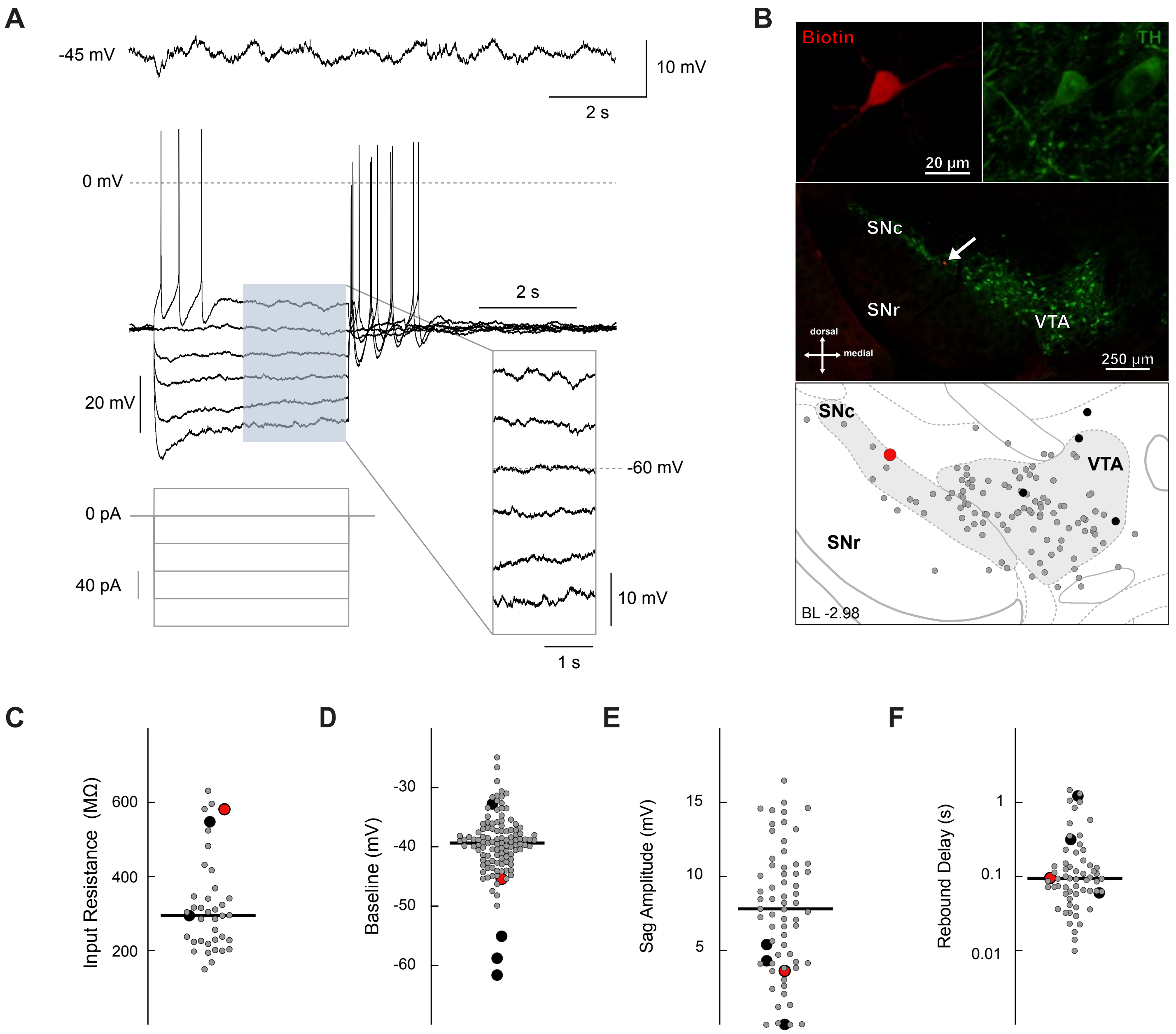
A
-45 mV
10 mV
2 s
0 mV
2 s
20 mV
-60 mV
0 pA
40 pA
10 mV
1 s
B
Biotin
TH
20 µm
SNc
SNr
VTA
dorsal
medial
250 µm
SNc
VTA
SNr
BL -2.98
C
Input Resistance (MΩ)
600
400
200
D
Baseline (mV)
-30
-40
-50
-60
E
Sag Amplitude (mV)
15
10
5
F
Rebound Delay (s)
1
0.1
0.01

Supplemental Figure 1. Silent and quiet dopamine cells are rare. **a.** Upper trace illustrates the hyperpolarized membrane potential of a dopamine neuron that did not fire spontaneous action potentials. Lower traces illustrate the dopamine neuron firing in response to current injection. **b.** Immunocytochemical identification locates the recorded neuron (arrow) within the SNc. The depiction (lower panel) shows the location of the recorded neuron (large red dot) in relation to all the recorded neurons in this study (black and gray dots). Black dots in B-F represent all cells that fired spontaneously at < 1 spike per minute. **c-f.** Input resistance (**c**), baseline membrane potential (**d**), sag amplitude (**e**), and rebound delay (**f**) of dopamine neurons that fired spontaneously at < 1 spike per minute. Grey dots indicate the same measurements for spontaneously active dopamine neurons.

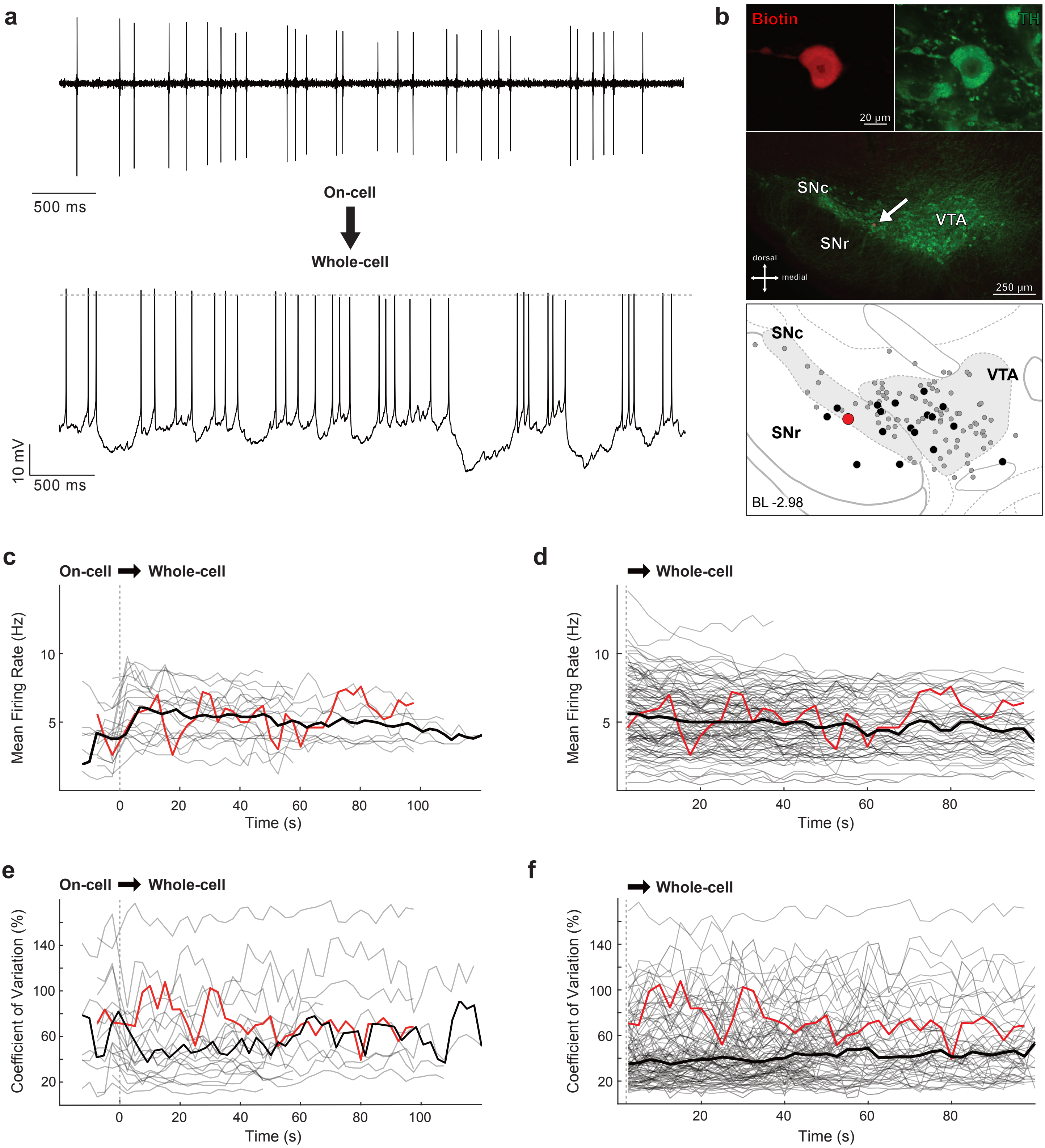
a
500 ms
On-cell
Whole-cell
10 mV
500 ms
b
Biotin
TH
20 µm
SNc
VTA
SNr
dorsal
medial
250 µm
SNc
VTA
SNr
BL -2.98
c
On-cell
Whole-cell
Mean Firing Rate (Hz)
Time (s)
d
Whole-cell
Mean Firing Rate (Hz)
Time (s)
e
On-cell
Whole-cell
Coefficient of Variation (%)
Time (s)
f
Whole-cell
Coefficient of Variation (%)
Time (s)

Supplemental Figure 2. *In vivo* whole-cell recording of dopamine neurons does not disturb firing rate or pattern of activity. **a.** Representative recording of spontaneous *in vivo* electrical activity from a neuron recorded in on-cell mode (upper trace) and then broken into the whole-cell configuration (lower trace) with similar firing rate and pattern. **b.** Immunocytochemistry locates the recorded neuron (arrow) within the medial region of the SNc. The depiction (lower panel) shows the location of the recorded neuron (large red dot) in relation to all the recorded neurons in this study (black and gray dots). Black dots represent all cells for which on-cell activity was recorded for comparison with whole-cell activity. **c.** Time series of mean firing rate during the transition from on-cell to whole-cell modes (vertical dashed line at time = 0; change in firing rate from on-cell to whole-cell returns to baseline within 70 seconds of breaking in = -19.86 (-14.86 to -4.36) Hz; n = 9, N = 9; p = 0.055; two-sided Wilcoxon Sign Rank test for paired data). **d.** Time series of mean firing rate for the first 100 seconds from all neurons recorded in this study (slope change in FR = -0.004; n = 108, N = 77). **e.** Time series of the coefficient of variation (CV) of firing during the transition from on-cell to whole-cell mode (vertical dashed line at time = 0; change in CV from on-cell to whole-cell returns to baseline within 20 seconds of breaking in = -11.75 (-17.7 to 7.26); n = 19, N =16; p = 0.184; two-sided Wilcoxon Sign Rank test for paired data). **f.** Time series of firing CV for the first 100 seconds from all neurons recorded in this study (slope change in CV = 0.003; n = 108, N = 77). **c-f.** Red lines represent the example neuron illustrated in A and B. Black lines represent the average of all lines for each panel.

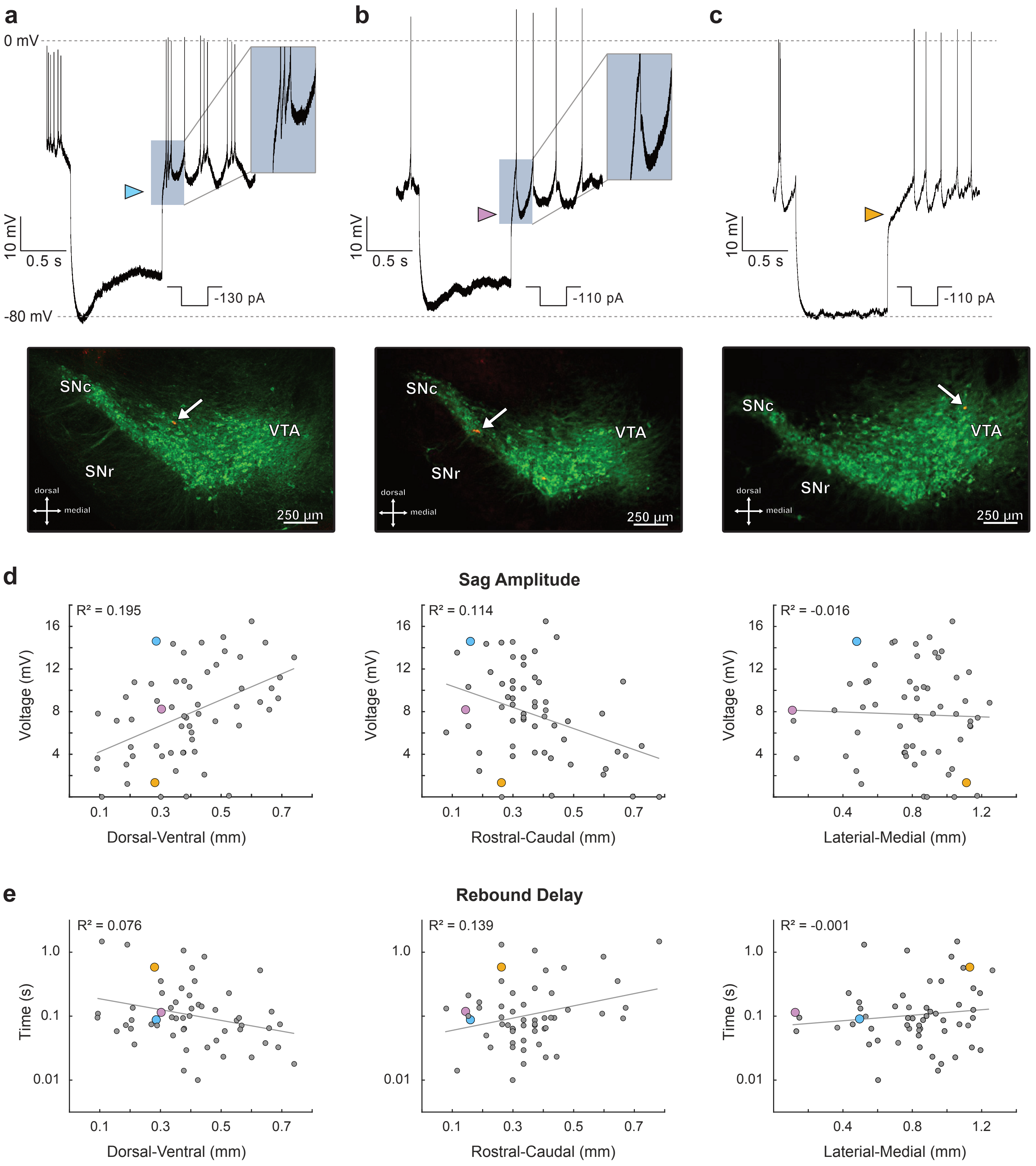

a
b
c
0 mV
-80 mV
10 mV
0.5 s
-130 pA
-110 pA
-110 pA
SNc
VTA
SNr
dorsal
medial
250 µm
d
Sag Amplitude
R² = 0.195
R² = 0.114
R² = -0.016
Voltage (mV)
Dorsal-Ventral (mm)
Rostral-Caudal (mm)
Laterial-Medial (mm)
e
Rebound Delay
R² = 0.076
R² = 0.139
R² = -0.001
Time (s)

Supplemental Figure 3. Sag and rebound delay are correlated with cell location. **a.** Example recording from an identified dopamine neuron with a large sag in response to hyperpolarizing current injection, and a short delay to the first spike upon removal of the hyperpolarization (blue arrowhead). **a-c.** The immunocytochemical images show the location (arrow) of the recorded neurons. **b.** Example recording from an identified dopamine neuron with a moderate sag in response to hyperpolarizing current injection, and a moderate delay to the first spike upon removal of the hyperpolarization (pink arrowhead). **c.** Example recording from an identified dopamine neuron with almost no sag in response to hyperpolarizing current injection, and a long delay to the first spike upon removal of the hyperpolarization (orange arrowhead). **d,e.** Relationship of cell location with sag amplitude (**d**) and rebound delay (**e**) for all neurons where the relevant protocols were carried out (sag amplitude at -80 mV = 7.8 (4.16 to 10.76) mV, n = 62; N = 53; rebound delay = 92.87 (58.5 to 151.05) ms; n = 58, N = 50). Both measures had a relationship with location in the dorsal-ventral and rostral-caudal planes. Colored dots in panels **d** and **e** correspond to the colored arrowheads in panels **a**-**c**

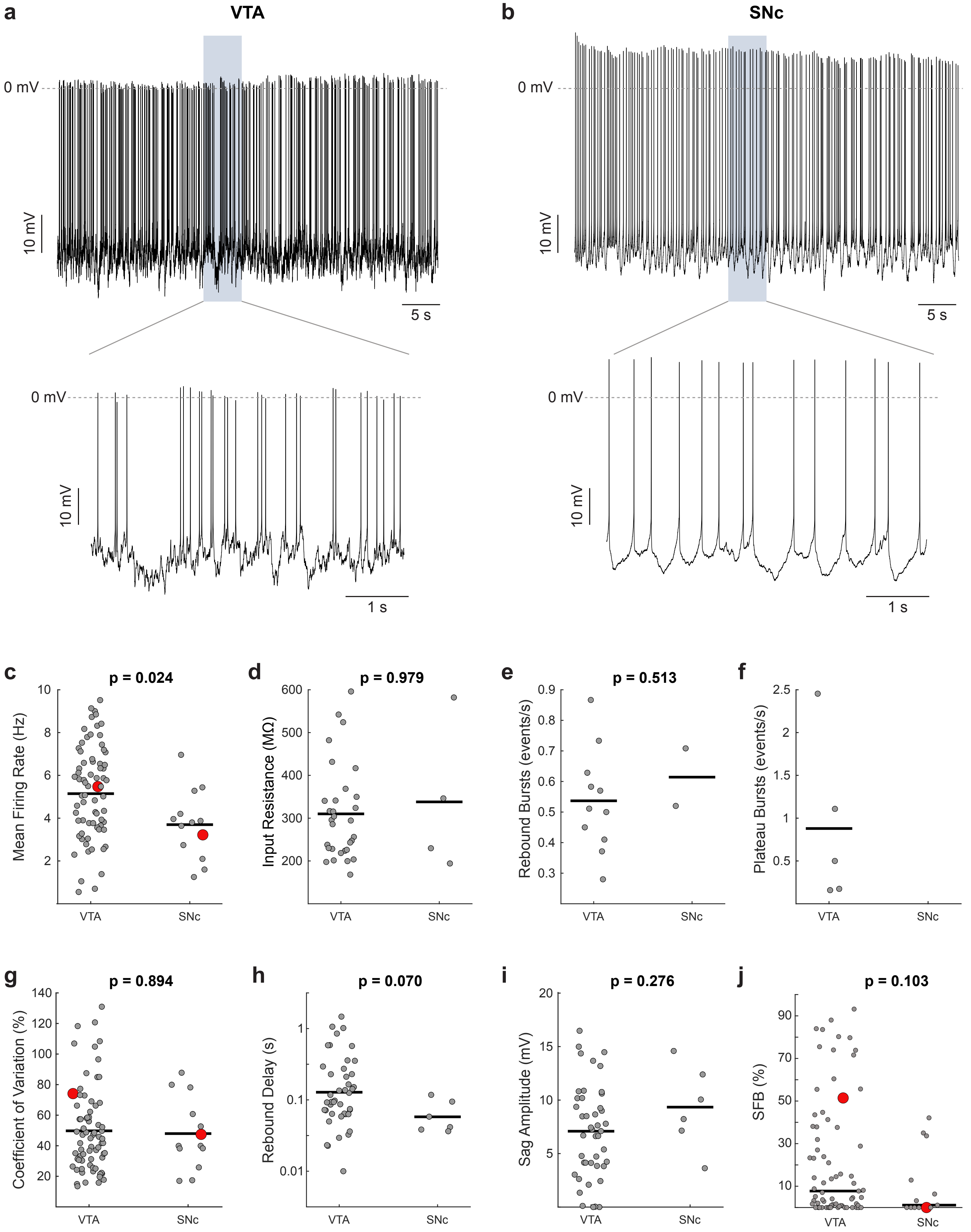

a
VTA
0 mV
10 mV
5 s
0 mV
10 mV
1 s
b
SNc
0 mV
10 mV
5 s
0 mV
10 mV
1 s
c
p = 0.024
Mean Firing Rate (Hz)
VTA
SNc
d
p = 0.979
Input Resistance (MΩ)
VTA
SNc
e
p = 0.513
Rebound Bursts (events/s)
VTA
SNc
f
Plateau Bursts (events/s)
VTA
SNc
g
p = 0.894
Coefficient of Variation (%)
VTA
SNc
h
p = 0.070
Rebound Delay (s)
VTA
SNc
i
p = 0.276
Sag Amplitude (mV)
VTA
SNc
j
p = 0.103
SFB (%)
VTA
SNc

Supplemental Figure 4. SNc and VTA dopamine neurons have similar electrophysiological characteristics *in vivo*. **a, b.** Traces recorded from identified VTA (**a**) and SNc (**b**) dopamine neurons. **c-f.** Mean firing rate (**c**), input resistance (**d**), rebound and plateau burst frequency (**e**, **f**), ISI CV (**g**), rebound delay (**h**), sag amplitude (**i**), and spikes fired in bursts as determined by the 80/160 ms criterion (**j**) of dopamine neurons located in the VTA and SNc. All analyses are Wilcoxon Rank Sum tests.

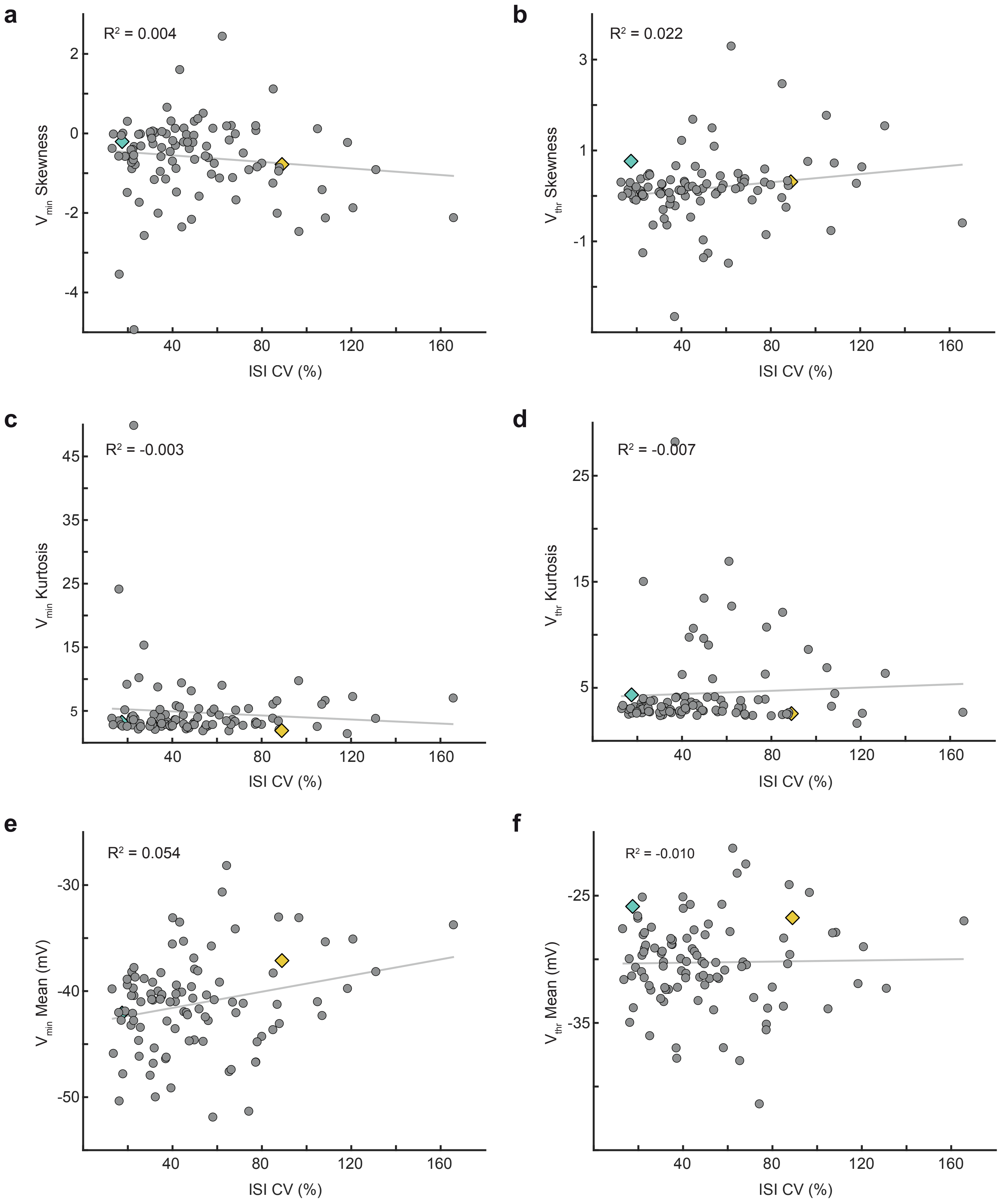

a
R² = 0.004
Vmin Skewness
ISI CV (%)
b
R² = 0.022
Vthr Skewness
ISI CV (%)
c
R² = -0.003
Vmin Kurtosis
ISI CV (%)
d
R² = -0.007
Vthr Kurtosis
ISI CV (%)
e
R² = 0.054
Vmin Mean (mV)
ISI CV (%)
f
R² = -0.010
Vthr Mean (mV)
ISI CV (%)

Supplemental Figure 5. Spike firing variability does not correlate with general measures of distribution. **a-f.** The coefficient of variation (CV) of the interspike intervals (ISI CV) correlates neither with skewness of the distributions for V_{min} (**a**) or V_{thr} (**b**), kurtosis of distributions for V_{min} (**c**) or V_{thr} (**d**), nor mean membrane potential of V_{min} (**e**) or V_{thr} (**f**; n = 97, N = 72). Cyan and yellow diamonds depict the values for the example cells illustrated in Figure 2 and the highlighted ISI CV in Figure 1f.

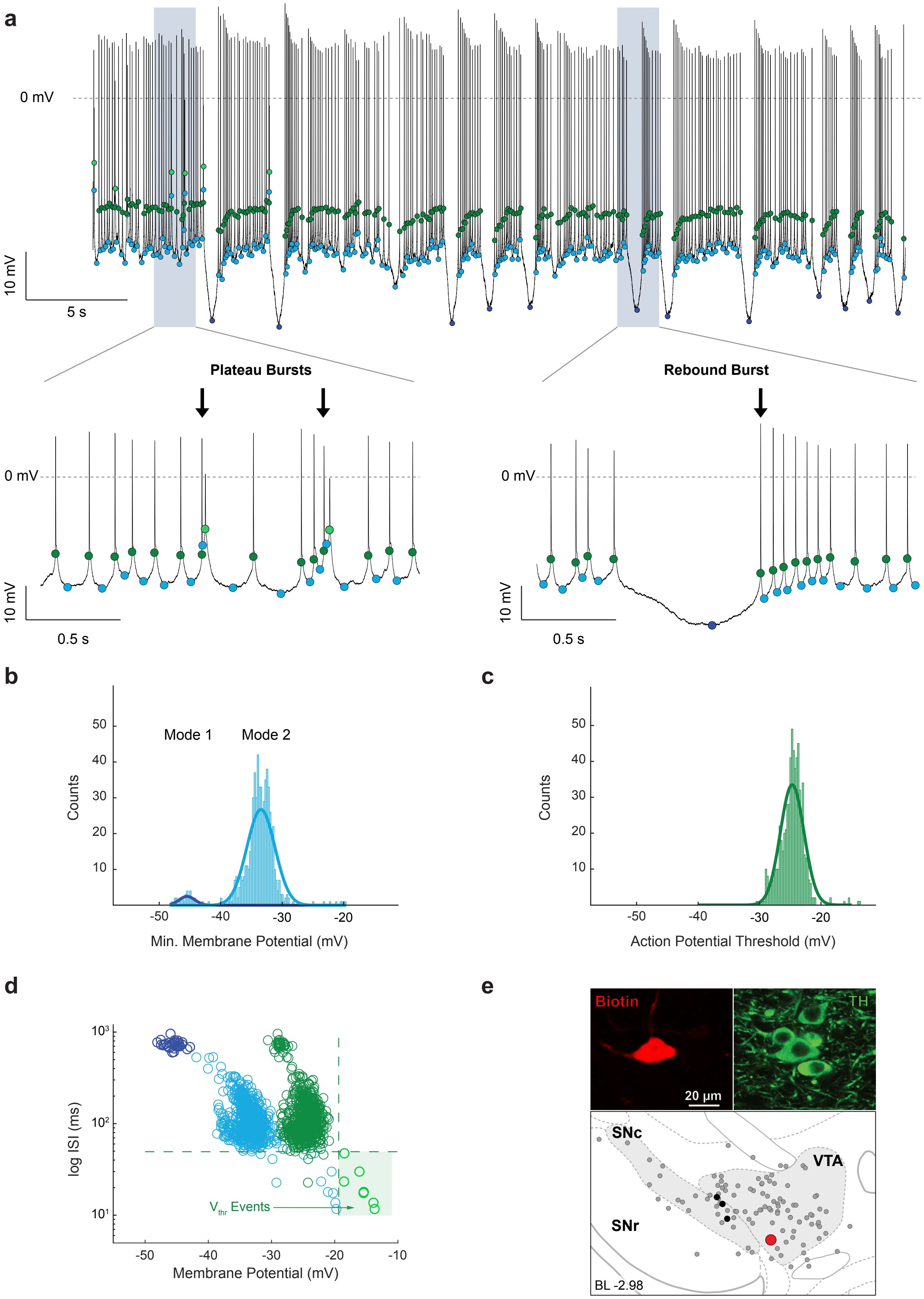

a
0 mV
10 mV
5 s
Plateau Bursts
Rebound Burst
0 mV
10 mV
0.5 s
0 mV
10 mV
0.5 s
b
Mode 1
Mode 2
Counts
Min. Membrane Potential (mV)
c
Counts
Action Potential Threshold (mV)
d
log ISI (ms)
V_{thr} Events
Membrane Potential (mV)
e
Biotin
TH
20 µm
SNc
VTA
SNr
BL -2.98

Supplemental Figure 6. Dopamine neurons can fire both rebound and plateau bursts during a single recording. **a.** Trace of a recording from an identified dopamine neuron. Lower traces illustrate examples of plateau bursts (left lower trace) and a rebound burst (right lower trace). **b, c.** Distributions for V_{min} (**a**), displaying a bimodal distribution; and V_{thr} (**b**) displaying a unimodal distribution. **d.** Scatter Plot of ISI versus V_{min} and V_{thr}. Green dashed lines indicate outlier identification in the lower left plane for V_{thr} with detected events in the shaded region. Dark blue circles identified from Mode 1 in panel **b**. **e.** Immunocytochemical identification and location (red dot).

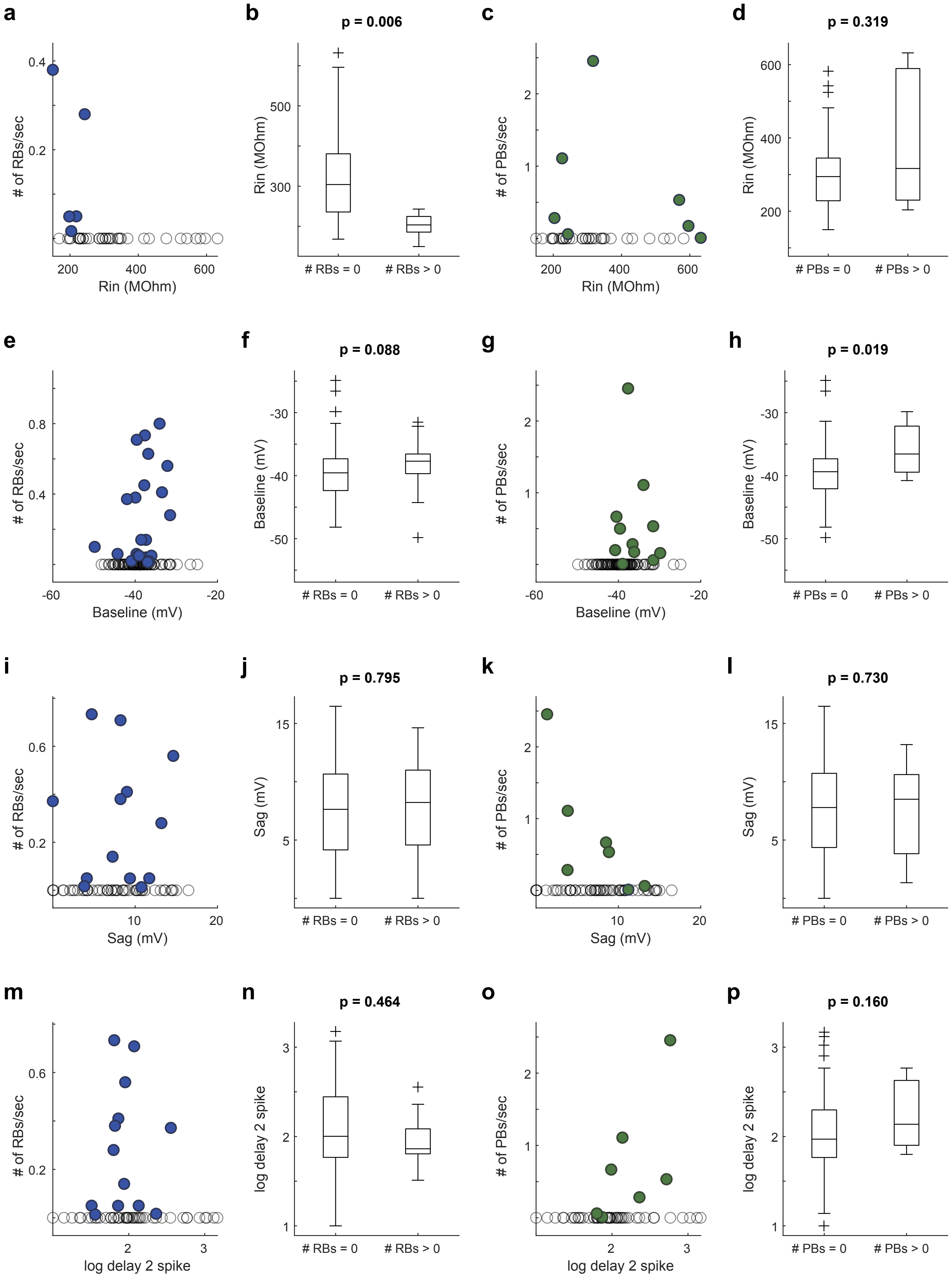

a
# of RBs/sec
Rin (MOhm)
b
p = 0.006
Rin (MOhm)
RBs = 0
RBs > 0
c
# of PBs/sec
Rin (MOhm)
d
p = 0.319
Rin (MOhm)
PBs = 0
PBs > 0
e
# of RBs/sec
Baseline (mV)
f
p = 0.088
Baseline (mV)
RBs = 0
RBs > 0
g
# of PBs/sec
Baseline (mV)
h
p = 0.019
Baseline (mV)
PBs = 0
PBs > 0
i
# of RBs/sec
Sag (mV)
j
p = 0.795
Sag (mV)
RBs = 0
RBs > 0
k
# of PBs/sec
Sag (mV)
l
p = 0.730
Sag (mV)
PBs = 0
PBs > 0
m
# of RBs/sec
log delay 2 spike
n
p = 0.464
log delay 2 spike
RBs = 0
RBs > 0
o
# of PBs/sec
log delay 2 spike
p
p = 0.160
log delay 2 spike
PBs = 0
PBs > 0

Supplemental Figure 7. Comparison of dopamine neurons with or without bursts. **a-d.** Input resistance of dopamine neurons with and without (rebound burst grey circles; n = 35, N = 33; plateau burst grey circles; n = 33, N = 30) rebound bursts (blue dots; **a**, **b**; n = 5, N = 5) or plateau bursts (green dots; **c**, **d** D). **e-h.** Baseline membrane potential of dopamine neurons with and without rebound bursts (**e**, **f**; rebound burst grey circles; n = 88, N = 66; plateau burst grey circles; n = 98, N = 73; blue dots n = 22, N= 21) or plateau bursts (**g**, **h**; n = 12, N = 12). **i-l.** Sag amplitude of dopamine neurons with and without rebound bursts (**i, j**; rebound burst grey circles; n = 47, N = 43; plateau burst grey circles; n = 52, N = 47; blue dots n = 13, N= 13) or plateau bursts (**k, l**; n = 8, N = 8). **m-p.** Delay to first spike from hyperpolarization of dopamine neurons with and without rebound bursts (**m, n**; rebound burst grey circles; n = 46, N = 42; plateau burst grey circles; n = 51, N = 46; blue dots n = 13, N= 13) or plateau bursts (**o, p**; n = 8, N = 8). Box plots are 25th (q1), 50th (q2) and 75th (q3) percentiles. Whiskers are q1 - 1.5 x (q3 - q1) and q3 + 1.5 x (q3 - q1). Points outside the whisker range are plotted individually. All comparisons done using a two-sided Wilcoxon Rank Sum test.

Supplemental Data Table 1

Table showing that there were no significant differences in firing rate and CV of ISI between various groupings of cells:

- Cell from animals belonging to sub-strains C57BL/6N vs C57BL/6J
- Cells from male vs female animals
- Recordings made using internal solution containing 40 nM vs 80 nM free calcium

All values are reported as median (25th quantile-75th quantile), and all p-values are reported for the Wilcoxon rank sum test. Upper-case letter ‘N’ indicates the number of animals and the lower-case letter ‘n’ indicates the number of cells in that grouping.

**Comparison type**	**FR (Hz)**			**CV**		
**C57BL/6N (n=90, N=64) vs C57BL/6J (n=20, N=15)**	**N**	**J**	**p-value**	**N**	**J**	**p-value**
	5.17 (3.56-6.96)	5.39 (3.61-6.62)	0.83	41 (26 - 64) %	49 (39 - 68) %	0.14
**Male (n=98, N=72) vs Female (n=12, N=7)**	**m**	**f**	**p-value**	**m**	**f**	**p-value**
	5.07 (3.56-6.58)	6.07 (4.38-7.2)	0.23	0.41 (0.26-0.66)	0.45 (0.41-0.58)	0.32
**[Ca^{++}] 40nM (n=7, N=5) vs 80nM (n=103, N=74)**	**40**	**80**	**p-value**	**40**	**80**	**p-value**
	4.25 (1.8-6.4)	5.27 (3.63-6.88)	0.33	0.45 (0.38-0.75)	0.42 (0.3-0.65)	0.72

Supplemental Data Table 2

Table comparing different measures taken from *in vivo* and *in vitro* whole-cell recordings, and *in vivo* juxtacellular recordings. All values are reported as median (25th quantile-75th quantile), and all p-values are reported for the Wilcoxon rank sum test.

**Input Resistance (MΩ)**		
**In Vivo Whole-cell (n = 42)**	**In Vitro Whole-cell (n = 71)**	**p-value**
295.2 (227.6-354.5)	443.1 (288.1-641.7)	<0.0001
**80/160 SFB (%)**		
**In Vivo Whole-cell (n = 110)**	**In Vivo Juxtacellular (n = 102)**	**p-value**
9.81 (0.755-38.5)	1.48 (0.113-9.30)	0.0007
**Firing Rate (Hz)**		
**In Vivo Whole-cell (n = 110)**	**In Vivo Juxtacellular (n = 102)**	**p-value**
5.25 (3.54-6.81)	3.16 (2.22-4.31)	<0.0001
**CV (%)**		
**In Vivo Whole-cell (n = 110)**	**In Vivo Juxtacellular (n = 102)**	**p-value**
42.5 (30.2-65.6)	49.7 (31.8-73.0)	0.1031

Supplemental Data Table 3

Table showing trajectories of repolarization speed (min dV/dt) across three types of spiking activity patterns. All values are reported as median (25th quantile-75th quantile), and all p-values are reported for the Wilcoxon rank sum test.

**Spiking pattern**	**min dV/dt spike 1 vs spike 2 (V/s)**		**min dV/dt spike 2 vs spike 3 (V/s)**	
	**Magnitude**	**p-value**	**Magnitude**	**p-value**
**Pacemaker**	0.003 (-0.0066 to 0.012)	0.38	-0.082 (8.95x10-5 to 0.017	0.19
	**Magnitude**	**p-value**	**Magnitude**	**p-value**
**Rebound burst**	-6.85 (-10.97 to -5.63)	0.002	2.87 (2.19 to 3.08)	0.002
	**Magnitude**	**p-value**	**Magnitude**	**p-value**
**Plateau burst**	0.53 (-0.2 to 1.77)	0.7	7.16 (5.34 to 8.31)	0.002
	**Magnitude**	**p-value**	**Magnitude**	**p-value**
**Mixed pattern rebound**	-3.91 (-4.73 to -3.17)	4.88×10^{-4}	1.29 (0.94 to 1.76)	4.88×10^{-4}
	**Magnitude**	**p-value**	**Magnitude**	**p-value**
**Mixed pattern plateau**	0.74 (0.043 to 1.38)	0.5	5.81 (0.28 to 11.33)	0.5
