# Supplementary material for: In vivo patch-clamp recordings reveal distinct subthreshold signatures and threshold dynamics of midbrain dopamine neurons
Source: Nat Commun. 2020 Dec 8;11:6286. doi: 10.1038/s41467-020-20041-2 (PMC7722714; doi:10.1038/s41467-020-20041-2)
Supplement: Supplementary file 3 — Reporting Summary [file 41467_2020_20041_MOESM3_ESM.pdf]

nature research

Corresponding author(s): Carlos A. Paladini
Last updated by author(s): 10/27/2020

Reporting Summary

Nature Research wishes to improve the reproducibility of the work that we publish. This form provides structure for consistency and transparency in reporting. For further information on Nature Research policies, see our Editorial Policies and the Editorial Policy Checklist.

Statistics

For all statistical analyses, confirm that the following items are present in the figure legend, table legend, main text, or Methods section.

n/a	Confirmed	
☐	☒	The exact sample size (*n*) for each experimental group/condition, given as a discrete number and unit of measurement
☒	☐	A statement on whether measurements were taken from distinct samples or whether the same sample was measured repeatedly
☐	☒	The statistical test(s) used AND whether they are one- or two-sided<br>*Only common tests should be described solely by name; describe more complex techniques in the Methods section.*
☐	☒	A description of all covariates tested
☐	☒	A description of any assumptions or corrections, such as tests of normality and adjustment for multiple comparisons
☐	☒	A full description of the statistical parameters including central tendency (e.g. means) or other basic estimates (e.g. regression coefficient) AND variation (e.g. standard deviation) or associated estimates of uncertainty (e.g. confidence intervals)
☐	☒	For null hypothesis testing, the test statistic (e.g. *F*, *t*, *r*) with confidence intervals, effect sizes, degrees of freedom and *P* value noted<br>*Give P values as exact values whenever suitable.*
☒	☐	For Bayesian analysis, information on the choice of priors and Markov chain Monte Carlo settings
☒	☐	For hierarchical and complex designs, identification of the appropriate level for tests and full reporting of outcomes
☒	☐	Estimates of effect sizes (e.g. Cohen's *d*, Pearson's *r*), indicating how they were calculated

Our web collection on statistics for biologists contains articles on many of the points above.

Software and code

Policy information about availability of computer code

Data collection	HEKA Patchmaster (version 1.2) and Axograph (version 1.7.2) for ephys data collection; Neuroinfo (MBF Bioscience, version 2019) for image collection.
Data analysis	Matlab (version 2018b and 2020a) for ephys data analysis, Neuroinfo (MBF Bioscience, version 2019) for image registration and cell localization.<br>The custom code used for analyzing this data, along with sample data and documentation has been uploaded to a Github repository at https://github.com/anandsku/DA_invivo.<br>The data used to generate the results are available at a Harvard Dataverse repository at https://dataverse.harvard.edu/dataset.xhtml?persistentId=doi%3A10.7910%2FDVN%2FQ9ZXLZ&version=1.0.

For manuscripts utilizing custom algorithms or software that are central to the research but not yet described in published literature, software must be made available to editors and reviewers. We strongly encourage code deposition in a community repository (e.g. GitHub). See the Nature Research guidelines for submitting code & software for further information.

Data

Policy information about availability of data

All manuscripts must include a data availability statement. This statement should provide the following information, where applicable:

- Accession codes, unique identifiers, or web links for publicly available datasets
- A list of figures that have associated raw data
- A description of any restrictions on data availability

The data that support the findings of this study are available from the corresponding author upon reasonable request.

Field-specific reporting

Please select the one below that is the best fit for your research. If you are not sure, read the appropriate sections before making your selection.

☒ Life sciences ☐ Behavioural & social sciences ☐ Ecological, evolutionary & environmental sciences

For a reference copy of the document with all sections, see nature.com/documents/nr-reporting-summary-flat.pdf

Life sciences study design

All studies must disclose on these points even when the disclosure is negative.

Sample size

No sample size calculation was performed. Sample size was determined by canvasing dopamine cell recordings from VTA and SNC so that both regions were sufficiently represented. This was determined by post-hoc examination of cell locations to canvas from lateral SNC through medial VTA.

Data exclusions

Some cells were excluded for poor access or short duration recordings. These included all experiments since criteria for data exclusion were measured prior to any manipulations.

Replication

The experimental findings were independently replicated by multiple researchers. This paper highlights the replication of the experimental findings as it is combination of Carlos Paladini, Kanako Otomo, and Jessica Perkins performing these experiments in two separate labs (Paladini and Roeper labs). Approximately half of the experiments were performed in the Paladini lab with the other half in the Roeper lab. Replication is also achieved with different calcium concentrations, mouse substrains, and sexes. Replication numbers are supplied in Supplemental Table 1.

Randomization

Blind in vivo whole-cell recordings ensure randomization of which type of cell, and specific location, is recorded. Mice used for recordings were selected randomly by a person not performing the recordings.

Blinding

In vivo whole-cell recordings are blind to the cell recorded until post-hoc identification and localization. Steady state calcium levels were blind to the experimenter.

Reporting for specific materials, systems and methods

We require information from authors about some types of materials, experimental systems and methods used in many studies. Here, indicate whether each material, system or method listed is relevant to your study. If you are not sure if a list item applies to your research, read the appropriate section before selecting a response.

Materials & experimental systems

n/a	Involved in the study	
☐	☒	Antibodies
☒	☐	Eukaryotic cell lines
☒	☐	Palaeontology and archaeology
☐	☒	Animals and other organisms
☒	☐	Human research participants
☒	☐	Clinical data
☒	☐	Dual use research of concern

Methods

n/a	Involved in the study	
☒	☐	ChIP-seq
☒	☐	Flow cytometry
☒	☐	MRI-based neuroimaging

Antibodies

Antibodies used

For the tyrosine hydroxylase (TH) staining, polyclonal rabbit antibody (catalog no. 657012, Merck, 1:500) was used as the primary antibody, and AlexaFluor-488 goat anti-rabbit IgG (catalog no. A11008, ThermoFisher, 1:1000) as the secondary antibody. Neurobiotin and biocytin were visualized with AlexaFluor-568 streptavidin conjugate (catalog no. S11226, ThermoFisher, 1:1000).

Validation

Millipore Sigma states on their website that Anti-Tyrosine Hydroxylase Rabbit pAb is validated in a broad range of species for use in Dot Blot, Immunoblotting, Immunofluorescence, Frozen Sections for the detection of Tyrosine Hydroxylase.

Animals and other organisms

Policy information about studies involving animals; ARRIVE guidelines recommended for reporting animal research

Laboratory animals

C57BL/6N (Charles River Laboratories and Janvier Labs) and C57BL/6J (Jackson Laboratories) mice (67 males and 7 females, aged 8-16 weeks) were used for the experiments. Mice were single-housed only after head-plate implantation. Relative humidity was maintained at 30-70%. Temperature was maintained at 68-69 degrees Fahrenheit.

Wild animals	The study did not involve wild animals.
Field-collected samples	The study did not involve samples collected from the field.
Ethics oversight	All experimental procedures were approved by the German Regional Council of Darmstadt (TVA 54-19c20/15-F40/28) or the University of Texas at San Antonio Institutional Animal Care and Use Committees.

Note that full information on the approval of the study protocol must also be provided in the manuscript.
